# Development of professional identity in medical students through interprofessional simulation: a qualitative study

**DOI:** 10.1186/s41077-026-00425-y

**Published:** 2026-02-21

**Authors:** Louis Fiander, Marie Bryce, Thomas Gale

**Affiliations:** 1 University of Plymouth, Plymouth, UK; 2 Centre for Applied Medical Education and Healthcare Workforce Research, Peninsula Medical School, University of Plymouth, Plymouth, UK

**Keywords:** Interprofessional, Simulation, Professional identity, Medical students

## Abstract

**Background:**

Professional identity development in doctors is associated with confidence, resilience and alignment with professional values. Empirical study has found that Interprofessional Simulation-based Education (IPSE) is associated with professional identity development in medical students but does not demonstrate *how*. An IPSE curriculum is in place within the University of Plymouth medical curriculum. Using reflexive thematic analysis of participating medical student's interview data, subsequently triangulated with theory from sociological and psychological literature, potential mechanisms of professional identity development through IPSE are discussed and implications for educational design explored.

**Methods:**

A qualitative approach was adopted utilising semi-structured interviews. Purposive sampling was used to select 10 final-year medical students undertaking the IPSE curriculum. Participants were asked about experiences contributing to their perceptions of professions, interprofessional learning and their experiences of the IPSE curriculum. Data were transcribed, coded and analysed using Thematic Analysis. Further interpretative analysis was then conducted using two theoretical perspectives: Social Identity Theory and Identity Theory.

**Main findings:**

Pre-IPSE experiences were found to have contributed to professional identity development in the participants studied. Participants’ perceptions of what it is to ‘be’ a doctor or nurse identified clear distinctions between groups, notably relating to role and status.

In performing as a ‘doctor’ and engaging with ‘nurses’ as ‘doctors’ through IPSE, participants experienced increased confidence and perceived competence in ‘being’ a doctor, and reformulated their understanding of roles and their approach to interprofessional practice. Positive developments were initially limited by challenges with inter-group communication and feelings of pressure that align with phenomena described in theoretical literature such as intergroup anxiety.

**Conclusions:**

This study reinforces that engagement in IPSE can develop the professional identity of final-year medical students. Mechanisms suggested by the data include strengthened role identity, reformulated conceptions of other professionals’ roles through tacit identity enactment, and the development of a superordinate professional identity shared with nursing students. Analysis of the data presented also gives insight into features of IPSE that may mitigate limiting factors of interprofessional learning occurring in-vivo such as a sociologically informed debrief. Implementing sociological models such as mutual intergroup differentiation may allay intergroup anxiety and status differentials, thus enabling further professional identity development.

## Background

Professional identity is defined in recent literature as “…how we perceive ourselves as professionals based on our attributes, beliefs, values, motives and experiences in relation to our profession” [1]. It is recognised amongst educational institutions, health institutions and professional regulators as a critical area of development for medical students as they transition into working professionals. The development of a professional identity in doctors is associated with confidence, resilience and alignment with professional values [1–3].

Theoretically, interprofessional simulation-based education (IPSE) is a highly suitable modality for professional identity development. It provides experiential learning with meaning to participants, as scenarios encountered reflect those to be faced in clinical practice by their future professional selves. It also inherently incorporates genuine collaboration with other professionals. The simulation debrief formalises this interprofessional learning, enabling reflection within and between groups, and the development of interpersonal relationships. This reflection, and the development of relationships are likely to have been initiated in the preceding simulation activity.

Empirically, the international literature supports the potential of IPSE as an effective means of promoting professional identity development amongst medical students [4–7], but crucially does not explore the psychological or sociological mechanisms behind this.

Research on this topic has predominantly been quantitative and there are fewer qualitative studies [8, 9], limiting the identification and development of meaningful implications for educational practice. Without understanding mechanisms of professional identity development, there is a risk that medical education curricula may fail to capitalise on professional identity development amongst medical students.

Two theories dominate the literature on identity; Social Identity Theory (SIT) of social psychologists, Tajfel and Turner [10], and Identity Theory (IT) attributed to sociologist, Stryker’s [11] development of symbolic interactionism. This study sought to analyse data from participants first inductively through thematic analysis, and then reconcile the results of this with the three broad bases for identity proposed by SIT and IT`: ‘social group’ or ‘social category’ (dominant in SIT), ‘role’ (dominant in IT) and ‘person’ (present but peripheral in both SIT and IT). Both SIT and IT perspectives were felt to be likely useful lenses, given the significance of both social and role related structures within the professional environment.

A qualitative analysis of how IPSE contributes to the development of professional identity in medical students allows sociological and psychological frameworks to be related to educational practice – providing recommendations for a curriculum which fosters professional identity development. This study seeks to qualitatively explore the mechanisms of professional identity development through IPSE in this population.

## Methods

### Ethical approval

Ethical approval was granted by the University of Plymouth, Faculty of Health Research Ethics Committee. All participants provided written informed consent for participation in this research and were made aware of their right to withdrawal.

### Pre-existent simulation curriculum

Final year medical students at the University of Plymouth participate in an IPSE curriculum. This entails six two hour-long immersive simulation sessions, spread variably over the course of one academic year. Three of these sessions are ‘interprofessional’. Within these sessions, teams containing three medical students and three final year nursing students undertake simulation of medical emergency scenarios. Participants are rotated so groups are different in each session. Scenarios are preceded by instructional pre-briefing and concluded by structured debriefing utilising the constructivist, Advocacy-Inquiry-based “TeamGAINS” [12] approach. All sessions are co-debriefed with faculty from nursing and medicine to create a safe learning environment for all participants. Facilitators are trained in simulation-based education and specifically in the TeamGAINS debriefing model in order to promote debrief quality [13]. The TeamGAINS model was chosen as the main focus is on collective team performance rather than individuals within the team. It incorporates ‘circular questioning’, encouraging inter-participant reflection and feedback [12].

### Approach

This study used a qualitative approach to gather rich, in-depth data to support a theoretically informed analysis. Sumner describes qualitative research as an exploration of “…the subjective meanings through which people interpret the world” [14]. Social Identity Theory (SIT) and Identity Theory (IT) both describe identity as a product of the subjective meanings that one attaches to oneself [10, 11]. The clear parallel highlights the necessity for a qualitative approach to exploring professional identity.

### Sampling

Final year medical students from the University of Plymouth – Plymouth Campus were selected as the sampling frame. A non-probability, purposive sampling methodology was used to recruit 10 participants from this frame. The first 10 participants who volunteered, met the conditions of the sampling frame, and had experience of the IPSE curriculum, comprised the initial sample. Upon LF’s analysis of the data collected from this sample, and subsequent analysis by TG and MB, the sample size was deemed adequate due to the degree of information power recognised [15]. The specificity of both the project aim and the participant group recruited, the high quality of the dialogue, and the rich corpus of psychological and sociological theory guiding data analysis, contributed to this in particular. As such, no further participants were recruited beyond the initial sample. The sample consisted of 7 male and 3 female students. Ages ranged from 22 to 31 at time of interview.

### Interviews

Individual, semi-structured, face-to-face interviews were conducted by LF. An interview guide, synthesised collaboratively by all authors was used (Appendix 1) and interviews were audio-recorded. In collecting data, “professional identity” was not explicitly discussed. This was to ensure participants did not feel alienated by this potentially unfamiliar concept [16]. Instead, questions focussed on accessible concepts contributing to professional identity such as changes in beliefs [1].

### Coding and thematic analysis

Recordings of all 10 interviews were manually transcribed by LF. The entire corpus was considered when conducting thematic analysis. A reflexive approach to coding was taken [17]. All coding was undertaken by LF facilitated by Nvivo 12 Pro software [18]. Codes were reviewed independently by both MB and TG. Coding and theming were iterative processes with codes concretised at a late stage of the thematic analysis, as new interpretations of meaning and perspective arose. Transcripts were reviewed upon any modification of codes to ensure that the whole corpus was coded consistently. After thematic analysis, data were interpreted in relation to two theoretical standpoints; IT and SIT. Triangulation of these interpretative perspectives with the researcher’s inductive analysis highlighted areas of consensus and inconsistency across theoretical perspectives, enabling more reflexive interpretation of the data.

### Reflexivity

LF was studying medicine at the host institution and knew the participants. Whilst this salient shared social group membership may have reduced the likelihood of inadequate rapport [19] or power dynamics affecting the data [20] it is important to recognise the potential effect of this relationship on the interview discourse. This was considered prior to interviews and helped guide the creation of a pre-amble and interview schedule designed not to lead or insinuate a “correct” response. Interviews were conducted formally, and participants assured of confidentiality throughout. TG is a practicing consultant anaesthetist with significant experience working in interprofessional teams and facilitating team-based simulations. MB is a social scientist with expertise in qualitative methodologies and clinical education research, and provided a non-clinical perspective.

## Findings

Findings revolved around three broad areas: experiences contributing to professional identity development prior to IPSE; general perceptions of what it is to ‘be’ a doctor or a nurse; and participants’ experiences of IPSE. Within each section, themes/sub-themes were identified relating to aspects of IT and SIT. Key data extracts relating to the aforementioned areas and divided by identified themes/subthemes are presented in Tables 1, 2, 3 and 4. The interviewee from whom the quotation is sourced is referenced in brackets, e.g. (i1).

**Table 1:** Age at time of interview and gender of participants

Participant Age	Gender of Participant
27	M
22	M
31	M
25	M
23	M
23	M
24	M
27	F
24	F
24	F

**Table 2:** Clinical experiences contributing to professional identity development prior to IPSE

Theme	Example quote (followed by quote reference)
Observation of medical and non-medical professionals’ practice	“[…] [nurses] had to go and ask the doctor a lot more question-wise and actually get prescriptions signed. So, even though they were running the same clinics […] they didn’t have the same level.”. (i10) “There was often this assumption [by doctors] that the doctors’ […] requests and things were perhaps a bit more important than some of the other things that, particularly on reception, we had to do.”. (i9)
Interprofessional interactions	“It was a bit more difficult […] in the first few years of medical school, […] it’s just about getting more confidence, so now I make the effort to introduce myself to everyone in all departments, and […] ask them questions […]”. (i2)
Observations of healthcare culture	“[…] the traditional way of looking at it, is […] your consultants at the top, then the junior doctors, then your nurses, then the healthcare assistants […] and then admin staff […]”. (i10) “I think the lower down the ranks you go, the more that [responsibility] shifts from being completely responsible, to being partially responsible, knowing that you have support from your consultant […]”. (i5) […] there is definitely a distance and almost a tribalism […] I’ve only seen one doctor sit in the nurses’ common room”. (i1)
Constraints of placement for Interprofessional learning	“[…] you see one side of it, or you see the other side of it, but you don’t really see the interaction, and I think that’s kind of where that learning’s going to come from […]” (i8) “We’re not very independent […] and the fact that things change a lot means that, four or five weeks just doesn’t feel like enough to get a grounding into that.” (i10) “[…] placement experience wise, it can be difficult I think sometimes, to really get an idea of […] what do the nurses do […] Being one-week placements, it’s hard to get a grip of it […]”. (i8)

**Table 3:** Perceptions of what it is to ‘be’ a doctor or a nurse

Theme	Example quote (followed by Interviewee number)
Roles of a doctor	“[…] people will look at you as a symbol of leader, authority, be that patients or be that other staff members […]”. (i8) “But I would say the role of a doctor is to ultimately take responsibility for patient safety.”. (i4) “[…] making a management plan for a patient, making them feel as if they’re in safe hands with someone who knows what they’re doing…” (i6)
Characteristics of a doctor	“I think confidence seems to be a big difference. The GPs […] stroll around, they do what they want, because […] they know what they’re doing.”. (i7) “[…good doctors] have to be willing to learn as well as willing to teach.”. (i10) “[…] people really underestimate how, how long we have to train for […] we make a lot of sacrifices […]”. (i6)
Role of a nurse	“[…] they’re constantly checking on them, constantly monitoring them, so they see a more evolving picture of that patient in hospital, compared to what I think a lot of doctors see […]”. (i3) “[…] making sure they’re getting to the loo, and getting fed, and eating properly, managing any pain they have […]”. (i6) "So, I would say the role of a nurse now is probably […] administration of doctors’ jobs which require someone who is clinically trained to do, when they’ve been handed off […]”. (i4)
Characteristics of a nurse	“There seems to be that closeness there […]”. (i9) “…when you break bad news, you’re encouraged to take a nurse with you… So that sort of empathy side…” (i5) “I do feel like nurses have a much better way of […] developing a rapport with patients that we don’t always do as doctors.” (i2)
Distinctions between doctors and nurses	“Nurses are seen, just inherently by our society, as lower, I want to say the word common? […] but when the doctor comes round, oh, you’ve got to be good.” (i1) “[…] medical training is a lot more theory, it’s a lot more years.” (i7) “I think a lot of people can see themselves as a care giver in general, and […] being more akin to a kind of a nursing role [than a doctor’s role] […]”. (i9)

### Experiences contributing to professional identity development prior to IPSE

Participants reported a mixture of experiences, from placements at medical school where situated learning is described, to work as Allied Healthcare Professionals outside of university. These experiences gave students opportunities to observe and partake in interprofessional communication, and witness aspects of healthcare culture – including ‘hierarchy’ and ‘tribalism’.

Making interprofessional connections made participants feel better able to learn and be involved in their placements. Through observation of professional interactions and clinical practice, participants developed an understanding of the practical dimensions of their own role and others’ roles.

However, whilst students reported being able to observe, they felt limited by the short time spent in each placement environment, and in their limited ability to ‘perform’ their prospective role as a doctor. When experiencing placements with different professional groups, they often felt siloed to one group, rather than having true interprofessional experiences.

### General perceptions of what it is to ‘be’ a doctor or a nurse

A doctor’s role was seen largely as that of a leader and problem solver, with minimal emotive language used to describe the role. Suggested characteristics of doctors were confidence, dedication and willingness to learn and teach. The role was described in a fashion consistent with IT; conveying the role of a doctor as “symbolic” of leadership and describing an ‘expectation’ of a doctor to lead the team and their patients. When asked about what they understood to be the role of a doctor, participants often gave their definition of a ‘good doctor’, speaking in value-loaded terms.

When describing nurses, participants used emotive vocabulary such as “comfort”, “caring” and “closeness”, far more so than when describing doctors. Perceived characteristics of nurses included being caring, empathic and good communicators. There was also a perceived nursing role of performing tasks *for* doctors.

Distinctions drawn between doctors and nurses focussed on pathways to qualification, length and construction of training, and the extent to which they rely on protocol in their work. Both these aspects reflected broader references to a status differential with nurses being perceived as below doctors.

### Participants’ experiences of IPSE

The IPSE curriculum undertaken presented interprofessional challenges, but also reaped significant developments in professional identity.

Perceptions of pressure and troublesome communication were widely reported by participants. Pressure was felt by participants due to a fear of appearing prejudiced against nurses and due to apprehension about making mistakes. This anxiety was felt on participants’ own behalf, and that of fellow medical students. Communication was challenging for participants specifically when interacting or choosing whether to interact with nursing student team members. This anxiety again precipitated difficulties when failure to recognise the medical problem within the scenario occurred. Participants demonstrated frustration towards nursing students in relation to this.

Despite these challenges, participants positively experienced the opportunity to ‘perform’ the role of a doctor, and with this reported increased confidence and perceived competence.

The interprofessional element of the IPSE enabled students to make social comparisons between groups and reassess formulations of capabilities, responsibilities, and status, including that of their own group, highlighting a progression over the longitudinal curriculum. This led to an understanding of each professional group’s distinct role, but also an understanding of mutual reliance.

All the IPSE scenarios were based around patients requiring emergency healthcare. This context was different to that in which some participants had previously observed nurses, and this enabled a novel understanding of the nursing role (Table 4).

**Table 4:** Participants’ experiences of IPSE

Theme	Sub-theme	Example quote (followed by quote reference)
Challenges presented by IPSE	Pressure	“You would expect us to be of a certain standard, […] someone who’s particularly poor at running the scenario, I think it’s going to give the wrong impression to the nurses.” (i4) “With nurses […] I was more scared […] I’d fear of being like a muppet. Sort of Imposter Syndrome […]”. (i1)
	Communication difficulties	“[…] when you’re asking a nursing student to do something […] you don’t want to seem like you are […] telling someone to do something, if that makes sense.” (i5) “However, in the debrief, […] one of the nurses said […] “oh I thought it was AF, and actually I felt like I couldn’t speak up.” […] I remembered asking, and she said: “Well I didn’t feel comfortable.” […] I felt a little bit hard done by, and I was a bit kind of annoyed […]” (i10)
	Disorganisation	“[…] freeze the scenario, and we were all talking amongst ourselves, and they were all talking amongst themselves, it was clear there was going to be two very different groups.” (i5)
Professional identity developments from IPSE	Increased confidence and perceived competence in ‘being a doctor’	“[…] seeing what people can and can’t do, has highlighted the ones especially that I can do, like cannulas, […] that you kind of take for granted that people can do.” (i7) "[…] as we sort of do more, and have more of an understanding of sort of our roles, and what we’re able to do, and, and what needs to be done […] I can see us becoming more confident and just go ahead and do it.” (i9)
	Negotiated understanding of what doctors and nurses do	“I think previously going in and thinking actually lots of this is our role but actually coming out of it and thinking actually we’re able to delegate more and offload, or be more effective in where we prioritise different things we do as doctors and actually give it to other professionals who are more than competent at doing it.” (i5)
	Improved interprofessional practice	"[…] we couldn’t do it without them, they couldn’t do it without us […] It’s not just doctors and nurses, we try and keep it in the sessions very mixed in all together. With the understanding of some people have different roles and different levels of competence […]”. (i7)

### Analysis and discussion

#### Experiences contributing to professional identity development prior to IPSE

Interprofessional interactions on placement and in the workplace contributed to participants’ professional identity development. These interactions bolstered participants’ sense of being ‘part of the team’. Empirical study has identified that a sense of belonging is closely associated with self-esteem and self-efficacy [21]. Perceiving oneself more as a ‘member of the team’ rather than as simply an observing student represents a development of professional identity [22].

However, simple observation *was* a mechanism of professional identity development for participants. Observation of hierarchy within hospitals allowed participants to categorise roles within their broader in-group [8] of ‘doctors’ into different hierarchical levels:

“I think the lower down the ranks you go, the more that [responsibility] shifts from being completely responsible, to being partially responsible, knowing that you have support from your consultant […]”. (i5).

This observation and categorisation, motivated by reduction of ‘self-related uncertainty’, allows medical students to more precisely understand their future role in a relational sense [23].

Understanding one’s role and that of others also allows one to better know the appropriate way to treat others [24]. IT would suggest that observations of positive practice contribute to medical students’ professional identity development via a mechanism where the observed positive perceptual input is compared to their pre-existing ‘doctor’ identity standards, verifying their professional identity.

‘Tribalism’ was also observed between professionals. This observation gave participants insight into how social and environmental factors within the healthcare system may precede conflict or perpetuate rivalry: “[…] there is definitely a distance and almost a tribalism […] I’ve only seen one doctor sit in the nurses’ common room”. (i1).

A SIT perspective suggests that witnessing others’ stereotyping of professional groups and how these stereotypes are engrained into culture may also contribute to the formation of stereotypes in medical students [23]. It suggests that the prototype of medical students’ professional in-group is informed by their experiences, and that medical students will adopt behaviours prescribed by this [23].

Despite the developments of identity discussed, participants felt that pre-IPSE placement experiences were too short-lived and too siloed to ‘act up’ as doctors and develop interprofessional understanding. Thistlethwaite’s [25] criticism of placement experiences as a source of interprofessional learning, given the potential for students to fall into distinct communities within the workplace, was reflected in participants’ accounts of pre-IPSE experiences. Experiential learning is seen as a key element of socialisation, allowing learners to develop ‘tacit’ elements of their professional identity [26]. Van den Broek et al. [27] found that successful performance of clinical tasks, and independent practice made final year medical students “feel like doctors”, enhancing the salience of their tacit identities. Participants’ comments on the limited time afforded to independent practice within the placement environment and their restricted ability to ‘act up’ indeed suggest shorter placements were inhibitory of professional identity development, and suggest a perceived need for a safe environment to act up, as is created by effective simulation-based education.

#### General perceptions of what it is to ‘be’ a doctor or a nurse

Participants’ unprompted integration of ‘the good doctor’ into discussion of the role of a doctor more generally suggests that, in defining themselves as future professionals, final year medical students develop a ‘good doctor’ prototype, as described by self-categorisation theory within SIT. Hogg et al.’s [24] concept of ‘meta-contrast’ suggests that in developing group prototypes, medical students will maximise intra-group similarities and intergroup differences. Members of the group seek to satisfy prototypes through adoption of accordant social behaviours, thereby developing their professional identity [24].

Empirical observations of intergroup stereotype formation found that when describing other groups, socio-emotive terminology was often used to describe those groups considered to be of lower status [28]. This suggests that participants’ stereotypes in relation to the role of nurses may be based on hierarchical assumptions. SIT would suggest that this demonstrates participants’ professional identification with doctors leading them to seek ‘positive distinctiveness’ from nurses in order to achieve self-enhancement. Whilst the stereotype of nurses being subservient to doctors is common [6], the unequal dynamic it engenders may potentially compromise patient safety [29].

Perceived status differential is recognised as conducive to intergroup prejudice [30], and thus obstructive to positive intergroup relations. Whilst status differential was not supported by participants, it was also not condemned. SIT may consider society’s reverence of doctors to be self-enhancing for medical students’ professional identity, motivating students to internalise the status differential between doctors and nurses.

Within SIT, decategorisation and re-categorisation are proposed as mechanisms of reducing such intergroup conflict [31]. These models suggest abolition of, rather than celebration of, subgroup identities such as ‘doctor’ or ‘nurse’, in favour of a shared superordinate identity such as ‘healthcare professional’. However, this is unlikely to be successful in the context of professional identities where subgroups are intimately tied to role obligations (such as doctors’ role obligations in signing off prescription medications, etc.). These role obligations mean that reducing the salience of subgroups may be compromising for potential shared tasks, or obstructive, as strengths of respective groups would not be optimally utilised.

Hewstone and Brown [32] propose Mutual Intergroup Differentiation (MID) as a means of reducing intergroup conflict, where contact alone fails to do so. MID advocates mutual acknowledgement of respective groups’ value and distinctiveness, whilst simultaneously recognising their inter-reliance and a shared superordinate identity. Empirical work by Richter et al. [33] identified that possession of both subgroup and superordinate identities in healthcare workers promoted productivity and reduced workplace intergroup conflict.

#### Participants’ experiences of IPSE

McCall and Simmons [34] describe in their work on IT, how others’ evaluations of one’s identity performance contribute significantly to the process of verifying identity. The stress of expectation described by participants likely relates to the identity-testing nature of their assumed external evaluation by the nursing students. The data suggest that the anticipated contact between the medical students (in-group [8]) and nursing students (out-group [8]) caused ‘intergroup anxiety’. Consequences of intergroup anxiety include categorisation and stereotyping which in turn may affect social behaviour in a potentially harmful way [35]. Indeed, apparent intergroup anxiety manifested as feeling pressured and as the blaming of out-group members in failure. These are risks of formalised intergroup contact in an educational setting, which mandate effective and targeted mitigation.

**Fig. 1:**
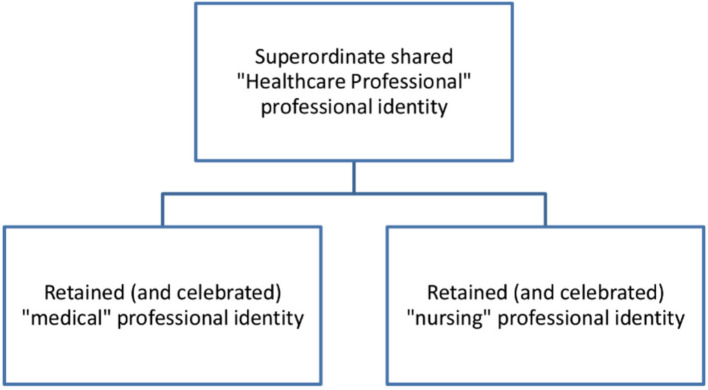
Diagram showing the MID model applied to the context of this IPSE

The reflective and exploratory nature of simulation debriefing lends itself to the addressing of troublesome intergroup relations [36]. The use of a validated debriefing framework has been found to correlate with improved debrief quality in simulation-based education [13]. Implementation of the MID model would require promotion of students’ mutual recognition of the value of, and differences between medical and nursing students, and the promotion of a superordinate ‘healthcare professional’ professional identity in combination with their subgroup ‘doctor or ‘nurse identities (Fig. 1).

With appropriate facilitator training, approaches used in current frameworks may be adapted to facilitate the implementation of intergroup conflict reduction strategies such as MID. The validated hybrid ‘TeamGAINS’ framework used in the IPSE curriculum described is amenable to promotion of MID. The Advocacy-Inquiry component allows for instructor led reflection on participants’ “taken-for-granted assumptions” whilst a combination of guided group self-correction and circular questioning facilitate an intra-group reflective process often not seen in traditional debriefing [12].

Whilst intergroup anxiety was an obstacle in some areas of the IPSE, significant identity development was observed in other areas. Perceptions of medical students’ roles within the IPSE were themed around leadership and problem-solving, much like their general perceptions of what it is to be a doctor. However, as well as identifying and consolidating their own distinct role, participants came to recognise that nurses too hold their own meritorious role, and that medical and nursing students were more effective in cooperation: “I think previously going in and thinking actually lots of this is our role but actually coming out of it and thinking actually we’re able to delegate more and offload, or be more effective in where we prioritise different things we do as doctors and actually give it to other professionals who are more than competent at doing it.” (i5).

These findings suggest that the longitudinal IPSE curriculum undertaken may in itself have facilitated MID, thus contributing to reduced intergroup conflict. Several aspects of the IPSE design may facilitate MID, including the shared objective requiring intergroup cooperation, and the division of tasks between groups—requiring the utilisation of each groups’ strengths.

Participants reported these transformational moments throughout the year-long IPSE curriculum. The longitudinal nature of the IPSE curriculum allows intergroup contact to be sustained, providing plentiful opportunity for interaction, and reducing intergroup anxiety [37]. Lave and Wenger [38] describe situated learning as a longitudinal process, and thus developments gained from workplace interactions, simulated or otherwise, may not be replicated through one-off or ad hoc interventions, such as those implemented in much of the relevant contemporary literature [4, 5, 7]*.*

## Further work

Whilst exploration of the nursing students’ perspectives of the same curriculum is beyond the scope of this work, an understanding of their experiences of the curriculum should also be explored. Not only might this provide useful insights into nursing students’ professional identity development, but it may also provide an alternative perspective in relation to medical students’ professional identity development. Understanding nursing students’ perspective may also prevent future IPSE redesign informed by this research from impairing the IPSE experience of nursing students.

Turner and Reynolds describe how categorisations are influenced by social context [39]. The ‘medical emergency’ context for interprofessional practice prescribed by this IPSE curriculum provided participants with insight into new aspects of nurses’ roles. Diversifying the context of simulation scenarios beyond common medical emergencies, should be considered in the future. Palliative scenarios, for example, may enhance the salience of different aspects of medical and nursing students’ role identities potentially providing new insights for both professional groups.

### Strengths and limitations

This study was conducted in one medical school, and data were collected from medical students, all of whom were in their final year before graduation. These factors mean findings are specific to the population intended to be studied but may limit the transferability of the data. Further, as aforementioned, only medical students were interviewed, with the views of nursing students not explored in this paper.

However, from 10 semi-structured interviews, a rich corpus of data was gathered. In-depth analysis of the entire corpus through thematic analysis followed by correlation with established psychological and sociological theory has produced meaningful implications for future educational design. Areas for further work are highlighted including exploration of nursing students’ perspectives and professional identity development, exploration of diverse simulation scenarios to broaden understanding of interprofessional roles, and the potential benefits of incorporating MID into IPSE to mitigate factors such as intergroup anxiety that hamper professional identity development.

## Conclusions

The importance of considering professional identity in pedagogical design is emphasised by the present study.

Experiences prior to partaking in IPSE were found to contribute to participants’ professional identity. However a perceived lack of opportunity to ‘perform’ inter-professionally in the tacit ‘doctor’ identity, leaves a gap in the curriculum for an intervention that can permit this safely.

This study demonstrates that IPSE can transform medical students’ understanding of the role of doctors and nurses, recognising both mutual reliance as well as their distinct important roles. It also demonstrates how IPSE may foster participants’ confidence and perceived competence in enacting their tacit doctor identities in an interprofessional setting.

The data presented suggest that medical students do perceive a status differential between medical and nursing professionals and that this may, initially, contribute to intergroup anxiety and thus difficult intergroup relations when working with nursing students through IPSE. This status differential should be addressed through IPSE so that detrimental intergroup dynamics can be mitigated in-vitro before they manifest in real-world practice.

Theoretical models such as MID which promote positive distinctiveness, interprofessional reliance and shared superordinate goals may be utilised to do this. Integrating the promotion of MID into validated debriefing frameworks such as TeamGAINS is feasible and, over the course of a longitudinal curriculum, may dismantle problematic intergroup factors including intergroup anxiety and negative stereotyping. a

Improvement of intergroup relations may itself be seen as a professional identity development, but will also perpetuate the other positive outcomes of IPSE such as confidence and understanding of multi-professional socio-structural roles.

Finally, the data suggest that professional identity development continued throughout the curriculum implying that ad hoc interventions, such as those described in much of the relevant contemporary literature, are limiting. Provided conditions facilitating positive engagement between groups are met, a longitudinal IPSE curriculum may further promote professional identity development.

## Data Availability

The datasets used and/or analysed during the current study are available from the corresponding author on reasonable request.

## Abbreviations

IPSE: Interprofessional Simulation-based Education
SIT: Social Identity Theory
IT: Identity Theory
MID: Mutual Intergroup Differentiation
